# Study of complex impedance spectroscopic properties of La_0.7−*x*_Dy*_x_*Sr_0.3_MnO_3_ perovskite oxides

**DOI:** 10.1098/rsos.172201

**Published:** 2018-11-14

**Authors:** I. Sfifir Debbebi, S. Megdiche-Borchani, W. Cheikhrouhou-Koubaa, A. Cheikhrouhou

**Affiliations:** 1 LT2S Lab, Digital Research Center of Sfax, Sfax Technopark, Cité El Ons, Route de Tunis, Km 9, Sfax. BP 275, Sakiet Ezzit, 3021 Sfax, Tunisia; 2 Higher Institute of Computer Science and Multimedia of Sfax (ISIMS), Technological Center of Sfax, BP 242, SakietEzzit, 3021 Sfax, Tunisia; 3 Laboratory of Spectroscopic Characterization and Optical Materials (LaSCOM), University of Sfax, Faculty of Sciences, BP 1171, 3000 Sfax, Tunisia

**Keywords:** impedance spectroscopy, ac conductivity, modulus, the non-overlapping small polaron tunneling, correlated barrier hopping

## Abstract

The dysprosium perovskite La_0.7−*x*_Dy*_x_*Sr_0.3_MnO_3_ (*x* = 0.00 [LSMO] and 0.03 [LDSMO]) compounds were prepared by the sol–gel reaction and characterized by the X-ray diffraction technique. The electrical conductivity and modulus characteristics of the system have been investigated in the temperature and the frequency range 311–356 K and 209–5 × 10^7^ Hz, respectively, by means of impedance spectroscopy. The ac and dc conductivities were studied to explore the mechanisms of conduction of LSMO and LDSMO. The insertion of a small amount of Dy^3+^ in the La-site of LSMO perovskite oxide increases the value of the activation energy from 0.2106 to 0.5357 eV and enhances electrical resistivity values for almost two orders of magnitude.

## 1. Introduction

The field of manganites with perovskite structure materials has experienced a boom in research activity in recent years due to the potential technological application of the so-called colossal magneto-resistance (CMR) behaviour [1,2]. Most of the manganites presenting the CMR effect have a paramagnetic–ferromagnetic (PM–FM) transition at the Curie temperature. Their electrical resistivity shows a semiconductor behaviour above *T*_C_ and a metallic behaviour below *T*_C_. The understanding of the CMR phenomenon, metallic behaviour and the strong FM interactions is generally based on the double-exchange (DE) model [3]. In this model, there is an exchange of electrons from neighbouring Mn^3+^ to Mn^4+^ ions through oxygen when their core spins are parallel and hopping is not favoured when they are anti-parallel. However, it was suggested that the DE model is not enough to explain the CMR phenomenon. Some authors suggested that other factors such as the Jahn Teller effect [4] and phase separation [5,6] are responsible for the behaviour observed in manganite.

However, recent observations of rich electrical properties in perovskites oxides LaMMnO_3_ have triggered renewed attention to this class of materials. Doping the insulating LaMnO_3_ material with the divalent ions causes the conversion of a proportional number of Mn^3+^ to Mn^4+^. It is believed that the interaction between pairs of Mn^3+^ and Mn^4+^ ions is responsible for the electrical properties in these manganese oxides [7]. Pairs of Mn^3+^ and Mn^4+^ can be controlled by changing the doping level or oxygen stoichiometry.

In our previous work, we have studied the dysprosium doping effects on the structural, magnetic and magneto-caloric properties of La_0.7−*x*_Dy*_x_*Sr_0.3_MnO_3_ with *x* = 0, 0.02 and 0.03 [8].

Thus, we have found that the magnetic measurements show a single ferromagnetic to paramagnetic transition and a decrease in Curie temperature with increasing Dy-amount, which can be explained by the DE interaction. Moreover, we have found a large magneto-caloric effect that was observed in the samples. We have also found that the obtained results indicate that the polycrystalline sample with *x* = 0.03 could be considered as a potential candidate for magnetic refrigeration applications at room temperature. According to the result obtained for La_0.67_Dy_0.03_Sr_0.3_MnO_3_ oxide, and for the continuation of our research, we are interested in this paper, in the study of the electric, dielectric properties and the conduction mechanism of the La_0.7−*x*_Dy*_x_*Sr_0.3_MnO_3_ (*x* = 0.00 and 0.03) system by means of impedance spectroscopy.

## 2. Experimental details

The Sol–gel method was used to synthesize the La_0.7−*x*_Dy*_x_*Sr_0.3_MnO_3_ (*x* = 0.00 and 0.03) manganites compounds. In a typical process, the stoichiometric amounts of La_2_O_3_, Dy_2_O_3_, SrCO_3_ and MnO_2_ with high purity (greater than 99.9%) were dissolved in a concentrated nitric acid solution with continued stirring at 80°C for about 4 h resulting in a transparent solution. After total dissolution, citric acid, a complexant agent and ethylene glycol, a polymerization agent, were added, then evaporated at 130°C to produce a gel. This latter was dried at 150°C to obtain a dark brown powder. Then it was heated to 300°C to remove the remaining organic and decompose the nitrates of the gel. In order to get the La_0.7−*x*_Dy*_x_*Sr_0.3_MnO_3_ (*x* = 0.00 and 0.03) nanoparticles, the sample was calcined at 600°C and 800°C with a rate of 1°C min^−1^ for 6 h respectively, with intermediate grinding between these calcined temperatures. The obtained powder was then pressed into pellets (of about 1 mm thickness under an axial pressure of 4 t for 2 min) and sintered at 900°C for 24 h to improve crystallinity.

The phase identification and structural analysis were performed using a ‘Panalytical X pert Pro’ diffractometer with Cu-K*_α_* radiation (*λ* = 1.5406 Á). The structural refinement was carried out by the Rietveld analysis of the X-Ray powder diffraction data with the help of FULLPROF software [9].

A pellet of about 0.503 cm^2^ surface and about 0.14 cm thickness was used for the electrical measurements. The pellet disc was coated with Ag paste to ensure good electrical contact. All electrical measurements of real and imaginary components of the impedance parameters (*Z*′ and *Z*′) were made over a wide temperature range (311–357 K) and frequency (209–5 × 10^7^ Hz) using a Tegam 3550 impedance analyser interfaced to a compatible computer.

## 3. Results and discussion

### 3.1. Powder X-ray analysis

The X-ray diffraction study, carried out in our previous work [8], confirms that the compounds La_0.7−*x*_Dy*_x_*Sr_0.3_MnO_3_ (*x* = 0.00 and 0.03) are a single phase with no measurable impurity phases. The refinement of the XRD patterns with the FULLPROF program shows that the sample is found to crystallize in the rhombohedral system with R-3c space group for the *x* = 0.00 and in the orthorhombic system with Pnma space group for the other sample. The powder XRD patterns and the refined parameters of the prepared compounds LSMO and LSDMO are presented in reference [8].

**Figure 1.**
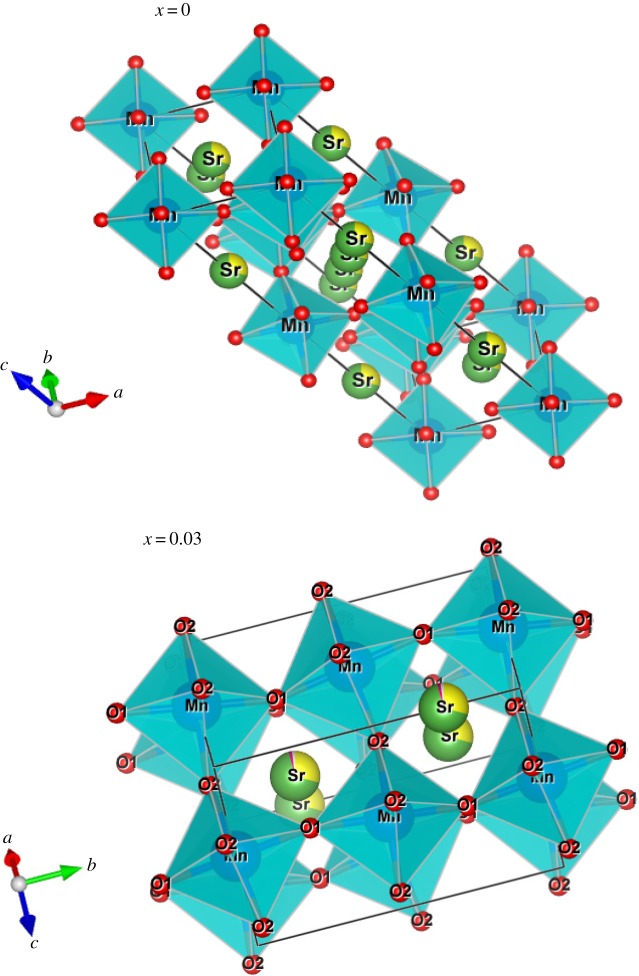
A projection along the *b*-axis and the plan of the La_0.7−*x*_Dy*_x_*Sr_0.3_MnO_3_ (*x* = 0.00 and 0.03) structures. The MnO_6_ groups are represented by solid and full octahedron, respectively.

Based on the Rietveld refinement, the unit cell was drawn, and the interionic separations were determined using the visualization software ‘Vesta’ [10]. Figure 1 shows a representative crystal structure and the MnO_6_ octahedron for both samples, which appears to be slightly distorted, in agreement with the previously reported results [11].

The atomic coordinates for La_0.7−*x*_Dy*_x_*Sr_0.3_MnO_3_ (*x* = 0.00 and 0.03) samples are listed in table 1, whereas important bond distances and bond angles associated with MnO_6_ and (La/Dy/Sr)O_12_ polyhedra are listed in table 2.

**Table 1.** Atomic coordinates for La_0.7−*x*_Dy*_x_*Sr_0.3_MnO_3_ (*x* = 0.00 and 0.03).

atoms	Wyckoff	*x*	*y*	*z*
LSMO				
La/Sr	6a	0.00	0.00	0.25
Mn	6b	0.00	0.00	0.00
O1	18e	0.4604	0.00	0.25
LDSMO				
La/Dy/Sr	4c	−0.0004	0.25	−0.0005
Mn	4b	0	0	0.5
O1	4c	0.4821	0.25	0.0594
O2	8d	0.2616	0.0256	0.7536

**Table 2.** Selected bond distances and angles for LSMO and LDSMO obtained from the refinement of X-ray diffraction data.

LSMO		LDSMO	
La/Sr–O (Å)	2.971(0)*3 2.534(9)*3 2.745(9)*6	La/Dy/Sr-O1 (Å) La/Dy/Sr-O2 (Å)	2.403(0) 2.671(8) 3.043(8) 2.862(9) 2.624(2)*2 2.862(3)*2 2.574(9)*2 2.902(5)*2
Mn–O (Å)	1.967(0)*6	Mn-O1 (Å) Mn-O2 (Å)	1.965(8)*2 2.003(0)*2 2.193(2)*2
Mn–O–Mn (°)	161.504(1)	Mn-O1-Mn (°) Mn-O2-Mn (°)	160.199(8) 136.860(2)
Sr–Sr (Å)	3.882(2)	Sr–Sr (Å)	3.863(9)

### 3.2. Impedance spectroscopy analysis

An impedance investigation of the ionic conductors over a wide frequency range has an advantage in that it allows the identification of charge transport processes in the grains and grain boundary of ceramics over a wide temperature range [12].

**Figure 2.**
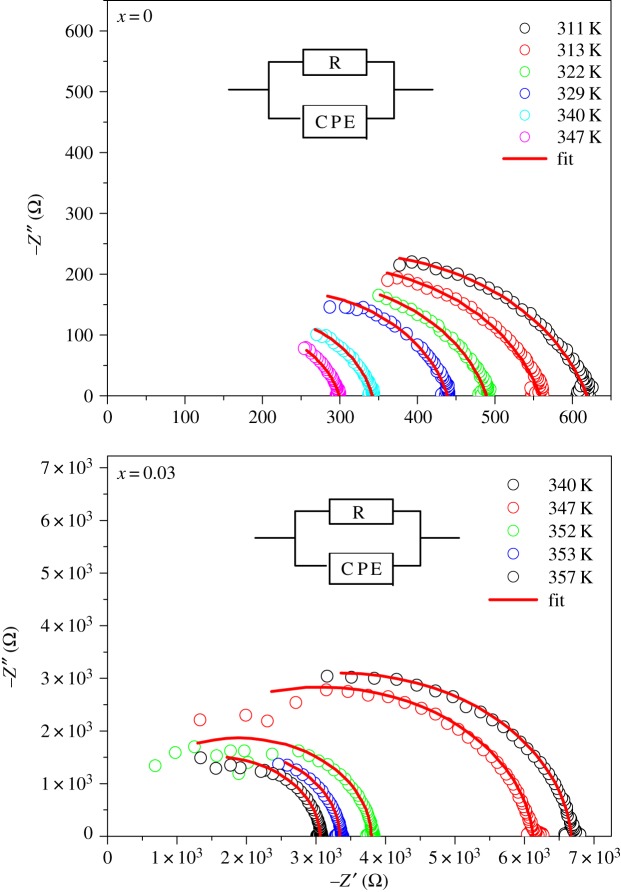
Nyquist plots with electrical equivalent circuit (inset) of La_0.7−*x*_Dy*_x_*Sr_0.3_MnO_3_ (*x* = 0.00 and 0.03) compounds at several temperatures.

Cole–Cole plots for LSMO and LDSMO at different temperatures are presented in figure 2. The data show a semicircle at all the temperatures. The bulk capacitance at the maximum of the semicircle can be determined using the relation: *ωR*_g_*C*_g_ = 1

The capacitance values for the frequency range semicircle are found to be in the range of 10^−12^F (pF) proving that the observed semicircle represented the bulk response of the system. A similar comportment is found in the literature for other ionic compounds [13].

From the Nyquist plots (figure 2) of LSMO and LDSMO samples, we can deduce that the electric resistance (the value of *Z*′ at −*Z*′′ = 0 at low frequencies) of these oxides increases with 3% of Dy doping by almost two orders of magnitude.

The impedance data were successfully modelled by an equivalent circuit, which is given in figure 2. This latter is composed of a parallel combination of resistance (*R*_g_) and capacitance (CPE_g_). The impedance of the capacity of the fractal interface CPE_g_ is given by the following:

3.1 $$Z=\frac{1}{Q(j\omega^{\alpha })},$$

where *Q* indicates the value of the capacitance of the CPE_g_ element and *α* the degree of deviation with respect to the value of the pure capacitor. This behaviour is typical of an ionic alloy conducting polycrystalline material [14].

The experimental data for the real (*Z*′) and imaginary (−*Z*″) components of the whole impedance were calculated from the theoretical expression established with equivalent circuit:

3.2 $$Z^{\prime }=\frac{R_{g}+R_{g}^{2}Q\omega^{\alpha }\cos(\alpha \pi /2)}{{\left(1+R_{g}Q\omega^{\alpha }\cos(\alpha \pi /2)\right)}^{2}+{{(R}_{g}Q\omega^{\alpha }\sin(\alpha \pi /2))}^{2}}$$

and

3.3 $$-Z^{\prime \prime }=\frac{R_{g}^{2}Q\omega^{\alpha }\sin(\alpha \pi /2)}{{\left(1+R_{g}Q\omega^{\alpha }\cos(\alpha \pi /2)\right)}^{2}+{{(R}_{g}Q\omega^{\alpha }\sin(\alpha \pi /2))}^{2}}.$$

**Figure 3.**
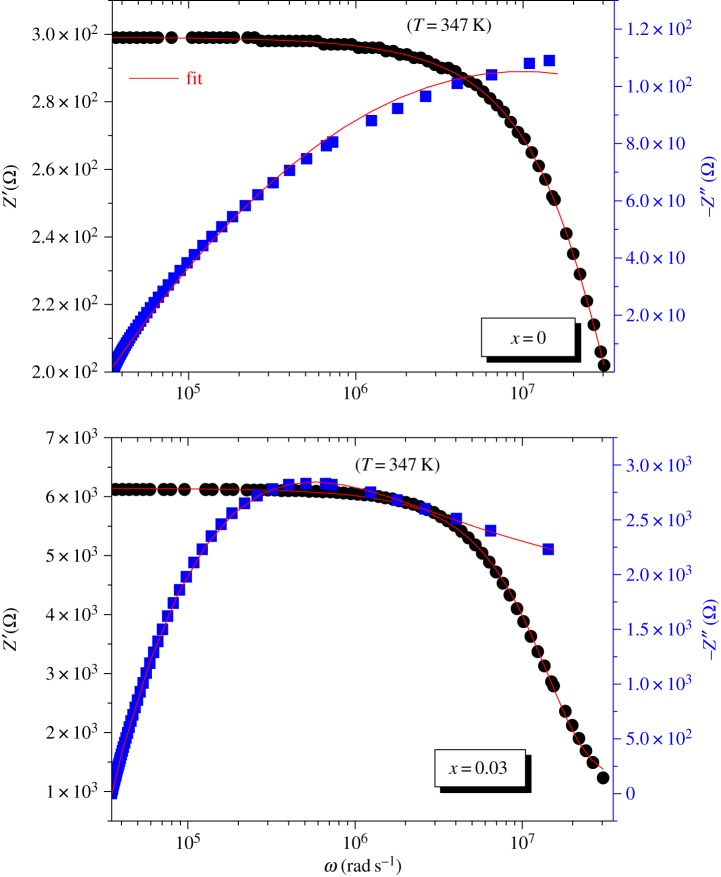
Frequency dependence of *Z*′ and −*Z*″ for LSMO and LDSMO perovkite oxides at 347 K.

*Z*′, –*Z*″ data measured at 347 K and their fits according to the above equations versus frequency are represented in figure 3 for LSMO and LDSMO compounds.

The good conformity of the calculated lines with the experimental data indicates that the suggested equivalent circuit describes the crystal–electrolyte interface reasonably well. Fitted values parameters for different temperatures are listed in table 3 for our LSMO and LDSMO perovskite oxides. As the temperature increases, the *R*_g_ value shifts towards a lower impedance value.

**Table 3.** Values of the electrical parameters deduced from the complex diagram for La0.7-xDyxSr0.3MnO3 (x = 0.00 and 0.03) compounds at several temperatures.

*T* (K) *x* = 0.00	*R*_g_ (Ω)	*Q* (nF)	*α*
311	564.6	1.738	0.820
313	564.8	1.738	0.820
322	494.1	1.495	0.829
329	442.4	1.129	0.84
333	386.5	1.078	0.849
340	344.2	0.970	0.855
347	300.3	0.806	0.866
352	273.8	0.670	0.876

*T* (K) *x* = 0.03	*R*_g_ (Ω)	*Q* (pF)	*α*
340	6504	29.40	0.974
347	6064	18.16	0.995
354	3347	14.38	0.989
357	3054	13.00	0.995

Figure 3 shows the frequency dependence of *Z*′ and −*Z*″, respectively, for the LSMO and LSDMO perovkite oxides at 347 K. From figure 3 we notice that as the frequency increases, −*Z*″ increases, whereas *Z*′ decreases. This trend continues up to a particular frequency in which −*Z*″ occupies a maximum value and in *Z*′ intersects.

### 3.3. Conductivity study

#### 3.3.1. DC conductivity

**Figure 4.**
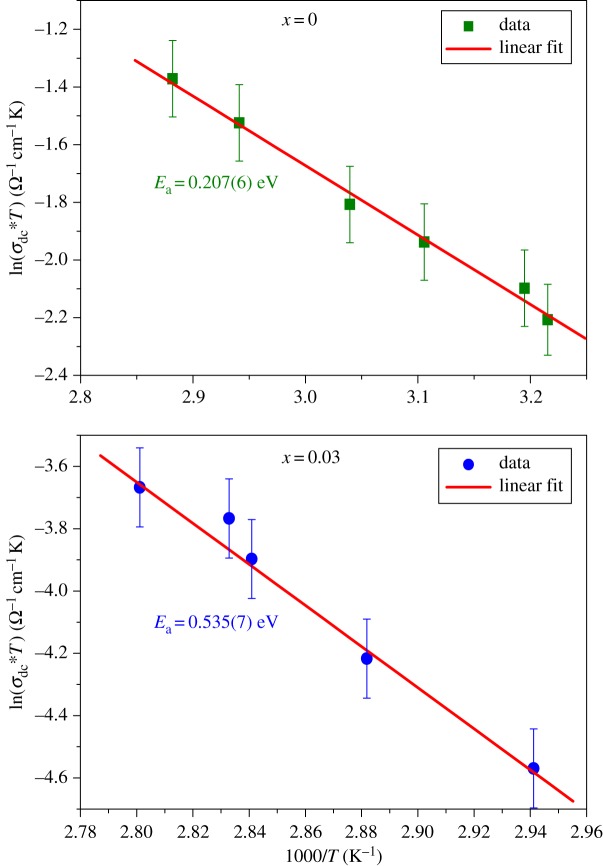
Variation of the ln(*σ*_dc_*T*) versus 1000/*T* for La_0.7−*x*_Dy*_x_*Sr_0.3_MnO_3_(*x* = 0.00 and 0.03) compounds.

The dc conductivity *σ*_dc_ reflects the steady state flow of current, its magnitude is often modified by the presence of electrode polarization or contact resistance. The dc conductivity of the grain is evaluated from the resistance *R*_g_ extracted from the equivalent circuit of our sample at different temperatures using the following relation:

3.4 $$\sigma_{\mathrm{dc}}=\frac{e}{S*R_{g}}.$$

where *S* is the electrolyte–electrode contact area, *e* is the thickness of the sample and *R*_g_ is the bulk resistance obtained from the intercept of the semicircular arcs observed at a higher frequency on the real axis (*Z*′). The temperature dependence of the conductivity ln (*σ*_dc_*T) versus 1000/*T* in grain effect of LSMO and LDSMO compounds are shown in figure 4. Arrhenius-type behaviour is observed and is described by:

3.5 $$\sigma_{\mathrm{dc}}*T=\sigma_{0}*\exp\left(\frac{-E_{a}}{k_{B}T}\right).$$

where *E*_a_ is the dc electrical activation energy, *σ*_0_ is the pre-exponential factor which including the charge carrier mobility and density of states and *k*_B_ is the Boltzmann constant.

The values of activation energy estimated from the Arrhenius plot of *σ*_dc_ for LSMO and LDSMO with respect to 1000/*T* are 0.210(6) eV and 0.535(7) eV, respectively. These values are higher than those obtained for other manganites, as shown in table 4 [15–17].

**Table 4.** Values of activation energy of our compounds as well as those for other oxides of the perovskite.

compounds	*E*_a_ (eV)	ref
La_0.7_Sr_0.3_MnO_3_	0.210	this work
La_0.67_Dy_0.03_Sr_0.3_MnO_3_	0.535	this work
Pr_0.7_Sr_0.3_MnO_3_	0.102	[15]
Pr_0.67_Sr_0.33_MnO_3_	0.030	[16]
La_0.6_Pr_0.1_Ba_0.3_MnO_3_	0.037	[17]

In the La_0.7−*x*_Dy*_x_*Sr_0.3_MnO_3_ (*x* = 0.00 and 0.03) series, we can correlate the activation energy of these materials, as indicated in table 5 with:

— the ionic radii of the cations in the A site of the AMnO_3_ perovskite oxide,
— the mismatch effect *σ*^2^ which is defined as [19,20]:
  3.6 $$\sigma^{2}=\sum_{i}y_{i}r_{i}^{2}-{\left\langle r_{A}\right\rangle }^{2},$$
and the bandwidth *W* calculated using the following formula [21]:
  3.7 $$W=\frac{\cos1/2(\pi -\langle \mathrm{Mn}-\mathrm{OMn}\rangle )}{{\left\langle \mathrm{Mn}-O\right\rangle }^{3.5}},$$
as reported earlier in mixed perovskite materials [18].

**Table 5.** Correlation of the activation energy *E*_a_ with the ionic radii 〈*r*_A_〉, the mismatch effect *σ*^2^ and the bandwidth *W*.

AMnO_3_ compound	*Z*	*V* (Å^3^)	*V*/*Z* (Å^3^)	〈*r*_A_〉 (Å)	*σ*^2^ 10^−2^ (Å^2^)	*W*	*E*_a_ (eV)	ref
La_0.7_Sr_0.3_MnO_3_	6	351.050	58.510	1.244	0.179	0.092	0.210	this work
La_0.67_Dy_0.03_Sr_0.3_MnO_3_	4	231.730	57.930	1.240	0.256	0.077	0.535	
Pr_0.5_Sr_0.5_MnO_3_	4	229.297	57.324	1.244	0.429	0.096	0.068	[18]
Pr_0.4_Gd_0.1_Sr_0.5_MnO_3_	4	227.583	56.895	1.237	0.570	0.092	0.107	

The electrical activation energy increases with the small addition of Dy content. This fact indicates that Dy doping enhances electronic localization. Such behaviour can be explained through the effect of both 〈*r*_A_〉 and the A site disorder quantified by the size variance (mismatch) *σ*^2^. In fact, the addition of Dy content inside the structure creates more disorder, which limits the DE mechanism. The reduction of 〈*r*_A_〉 induces a decrease of *e*_g_ electron bandwidth, which reduces electronic hopping. A similar result was reported for La_0*.*5−*x*_Gd*_x_*Sr_0*.*5_MnO_3_ samples [22].

One can note that *E*_a_ increases with Dy doping, which favours charge localization and makes electron hopping harder. This fact confirms that the addition of Dy content reduces the DE mechanism. This result is in good concordance with magnetic properties [8]. In fact, with increasing Dy content, the Curie temperature *T*_C_ decreases significantly (from 319 K for LSMO to 264 K for LDSMO), indicating a weakening of the DE interactions.

#### 3.3.2. AC conductivity

The measurement of the ac response provides important information about the conduction process in the materials. The ac conductivity was calculated using the relation [23]:

3.8 $$\sigma_{\mathrm{ac}}=\frac{e}{S}*\frac{Z^{\prime }}{Z^{\prime^{2}}+{Z^{\prime \prime }}^{2}},$$

where *S* is the cross-sectional area of the electrode deposited on the sample and *e* is the thickness of the pellet.

**Figure 5.**
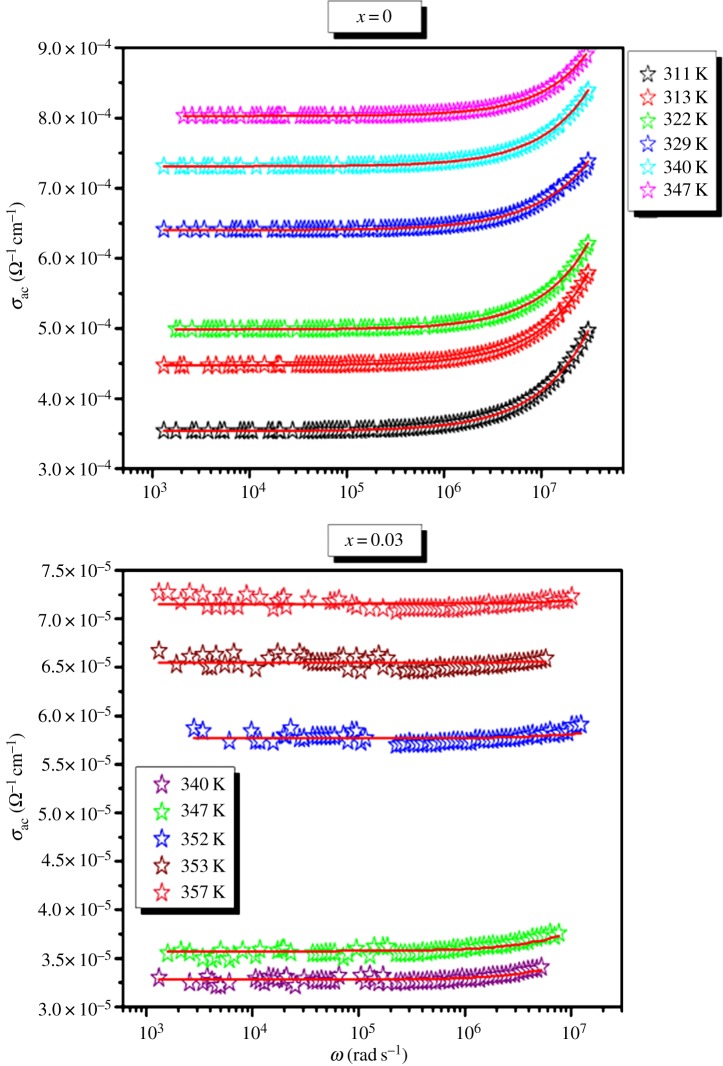
Angular frequency dependence of the ac conductivity at various temperatures of the La_0.7−*x*_Dy*_x_*Sr_0.3_MnO_3_ (*x* = 0.00 and 0.03) compounds.

The frequency dependence of ac conductivity at various temperatures for the LSMO and LDSMO are shown in figure 5. It is obvious that the addition of Dy decreases the conductivity for at least one order of magnitude. This behaviour indicates that Dy^3+^ ions block the displacement of Sr^2+^, and LDSMO becomes more resistive than LSMO.

The conductivity is noted to have dispersion at all frequencies in both samples. The phenomenon of the conductivity dispersion is generally analysed using Jonscher's law [24,25]:

3.9 $$\sigma_{\mathrm{ac}}=\sigma_{\mathrm{dc}}+A\omega^{s},$$

where *σ*_dc_ is the direct courant conductivity of the sample, *A* is a constant for a particular temperature which determines the strength of polarizability, and *s* is the power exponent where 0 < *s* < 1. The *s* represents the degree of interaction between mobile ions with the environments surrounding them, and *A* determines the strength of polarizability. Jonscher's law has been applied to many materials to analyse the ac conductivity behaviour in glasses and amorphous semiconductors [26–34]. This characteristic feature is well known in the disordered system.

Several theoretical models based on the relaxation caused by the hopping or tunneling of electrons or atoms between equilibrium sites have been developed to explain the frequency and temperature dependence of ac conductivity and the frequency exponent, s(T, *ω*) [35]. In this context, Gzaiel *et al*. [36] and Ben Bechir *et al*. [37] showed the behaviours of the exponent *s* and the corresponding conduction mechanism.

These models were more particularly developed to understand the mechanisms of electric conduction in disordered materials [38].

**Figure 6.**
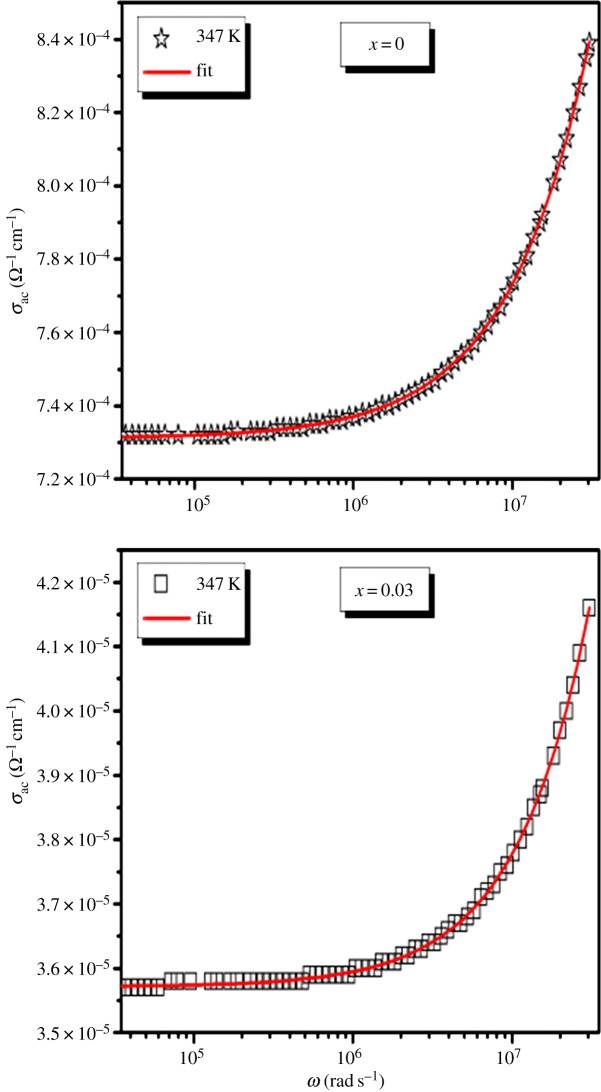
Angular frequency dependence of the ac conductivity at 347 K of the La_0.7−*x*_Dy*_x_*Sr_0.3_MnO_3_ (*x* = 0.00 and 0.03) compounds.

**Figure 7.**
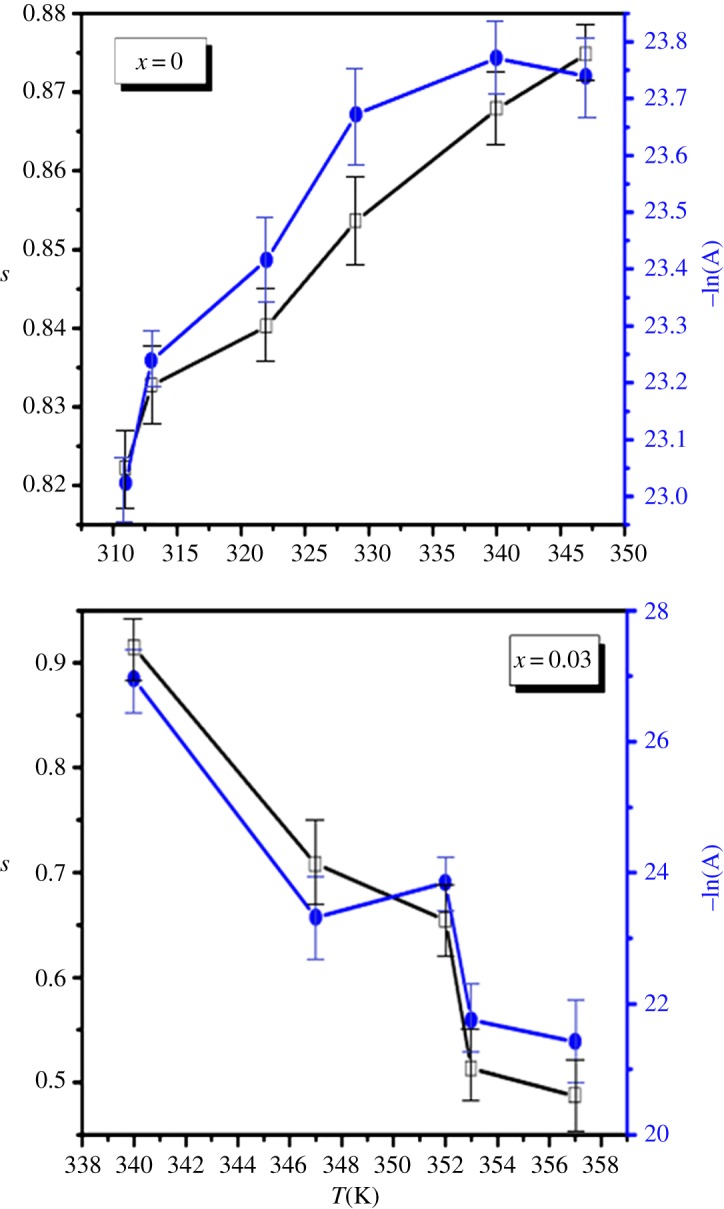
Variation of -LogA and s versus temperature for LSMO and LDSMO oxides.

The above equation (3.6) has been used to fit the ac conductivity data. In order to see better the conformity between the experimental and theoretical curves of frequency dependence of ac conductivity, we have reported in figure 6 *σ*_ac_(*ω*) curves at 347 K for our compounds. In the fitting procedure, *A* and *s* values have been varied simultaneously to get the best fits for LSMO and LDSMO oxides as shown in figure 7.

Since the values of exponent s lie in the range 0.48–0.91 for LSMO and 0.87–0.82 for LSDMO, the correlation motion is sub-diffusive and indicates a preference on the part of ions that has hopped away to return to where it started [39]. Jonscher [40] has shown that a non-zero *s* in the dispersive region of conductivity is due to the energy stored in the short-range collective motion of ions. A higher value of *s* value implies that large energy is stored in such collective motions. In this work, *s* increases with temperature for the parent compound LSMO (figure 7), suggesting that the non-overlapping small polaron tunneling (NSPT) [41] model is the appropriate one to characterize the electrical conduction mechanism in this sample. The Sr^2+^ ions move in the tunnel-like cavities along [111] direction as shown in figure 1. However, for the substituted LDSMO, The correlated barrier hopping (CBH) model, in which the exponent s decreases with increasing temperature as shown in figure 7, may be the most appropriate model [23,25].

#### 3.3.3. Theoretical investigation of conduction mechanism

##### 3.3.3.1. The NSPT model (*x* = 0.00: LSMO)

According to the NSPT model, the ac conductivity and the exponent *s* are given by [42,43]:

3.10 $$\sigma_{\mathrm{ac}}=\frac{{\left(\pi e\right)}^{2}K_{B}T\alpha^{-1}\omega {\left[N(E_{F})\right]}^{2}R_{\omega }^{4}}{12}$$

and

3.11 $$s=1+\;\frac{4K_{B}T}{W_{M}+K_{B}T\mathrm{Ln}(1/\omega \tau_{0})}$$

where

3.12 $$R_{\omega }=\frac{1}{2\alpha }\left[\mathrm{Ln}\left(\frac{1}{\omega \tau_{0}}\right)-\frac{W_{m}}{K_{B}T}\right].$$

From the above equations, *α*^−1^ is the spatial extension of the polaron, *R_ω_* is the tunneling distance, *N*(*E*_F_) is the states density near the Fermi level and *W*_m_ is the polaron hopping energy.

**Figure 8.**
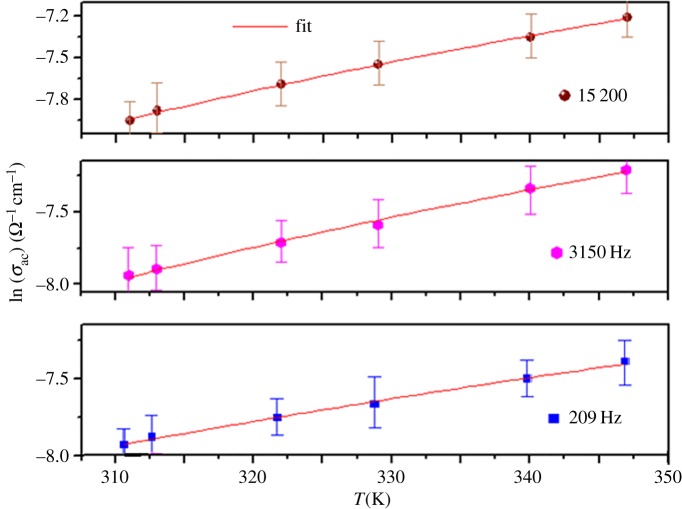
Temperature dependences of *σ*_ac_ at different frequencies of LSMO.

The variation of the ac conductivity (ln(*σ*_ac_)) as a function of 1000/*T* at different frequencies for La_0.7_Sr_0.3_MnO_3_ is given in figure 8. The calculated fitting parameters are collected in table 6.

**Table 6.** Parameters used for NSPT and CBH model fitting respectively for LSMO and LDSMO samples.

	La_0.7_Sr_0.3_MnO_3_ NSPT model			La_0.67_Dy_0.03_Sr_0.3_MnO_3_ CBH model	
frequency (Hz)	*α* (Å^−1^)	*N* (*E*_F_) (eV^−1^cm^−1^)	*W*_m_ (eV)	*N*_T_ (E^20^) (cm^−3^)	*W*_m_ (eV)
209	1.521(2)	1.520(1)E + 15	0.330(1)	2.241(4)	0.643(4)
3150	1.760(1)	9.511(1)E + 14	0.311(2)	1.980(1)	0.610(2)
15 200	1.832(4)	8.250(2)E + 14	0.300(3)	1.251(1)	0.651(5)

**Figure 9.**
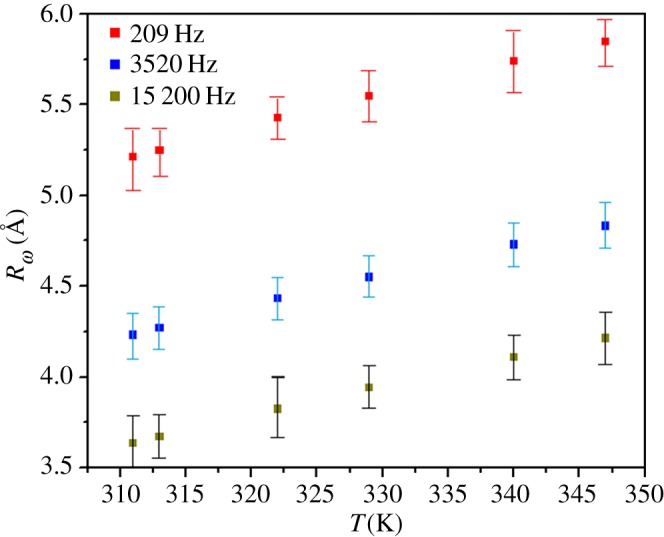
The temperature dependence of *R_ω_*(Å) of LSMO at different indicated frequencies.

The variation of *R_ω_* with the temperature at different frequencies is given in figure 9. It is clear that *R_ω_* decreases with frequency and it is in the order of the interatomic spacing, as shown in table 3.

##### 3.3.3.2. The CBH model (*x* = 0.03: LDSMO)

This model is called the CBH, in which the charge carrier hops between the sites over the potential barrier separating them. This model was first developed by Pike [44] for single-electron hopping and has been extended by Elliot [45] for two simultaneous electrons hopping.

The ac conductivity is determined by [46]:

3.13 $$\sigma_{\mathrm{ac}}=\frac{n\pi^{2}NN_{p}\varepsilon^{\prime }\omega R_{\omega }^{6}}{24}$$

where *n* is the number of electrons hopping (the density of pair sites is *n* = 1 for the single polaron case and *n* = 2 for the bipolaron case), *NN*_p_ is proportional to the square of the concentration of states and *ɛ*′ is the dielectric constant. *NN*_p_ is given by:

$NN_{P}=1/2\;N_{T}^{2}$ for bipolaron hopping and $NN_{P}=1/4N_{T}^{2}\exp(-U_{\mathrm{eff}}/2k_{B}T)$ for single polaron hopping, where *U*_eff_ is the effective correlation energy (correlation between electrons and phonons).

*R_ω_* is the hopping length given by the relation [47]:

3.14 $$R_{\omega }=\frac{e^{2}}{\pi \varepsilon^{\prime }\varepsilon_{0}[W_{M}-K_{B}T\ln(1/\omega \tau_{0})]}$$

where *k*_B_ is the Boltzmann constant, *T* is the absolute temperature, *W*_M_ is the binding energy, *ω* is the angular frequency and *τ*_0_ is the characteristic relaxation time, which is in the order of atom vibrational period *τ*_0_ = 10^−13^ s.

In this model, the exponent s is expressed as:

3.15 $$s=1-\;\frac{6K_{B}T}{W_{M}-K_{B}T\ln(1/\omega \tau_{0})}.$$

For first approximation (for large values of $W_{M}/K_{B}T$), the exponent *s* becomes expressed by:

3.16 $$s=1-\frac{6K_{B}T}{W_{M}}.$$

The binding energy *W*_M_ is defined as the energy required to move an electron completely from one site to another.

**Figure 10.**
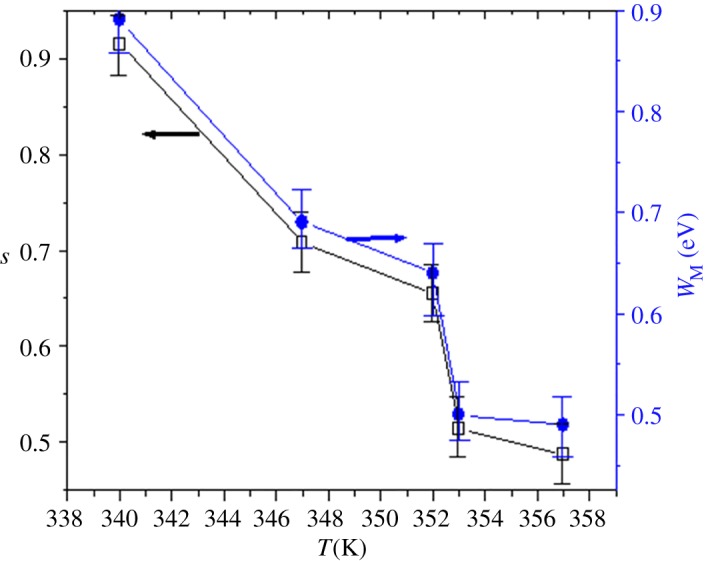
Temperature dependence of the *s* exponent and the binding energy *W*_M_ for LDSMO.

Values of *W*_M_ are calculated from equation (3.16) and shown in figure 10. *W*_M_ decreases with temperature, which corresponds to the decrease in the *s* exponent. So, the number of free carriers which can jump over the barrier will be reinforced. Consequently, this behaviour confirms that the *σ*_ac_ rises with temperature.

**Figure 11.**
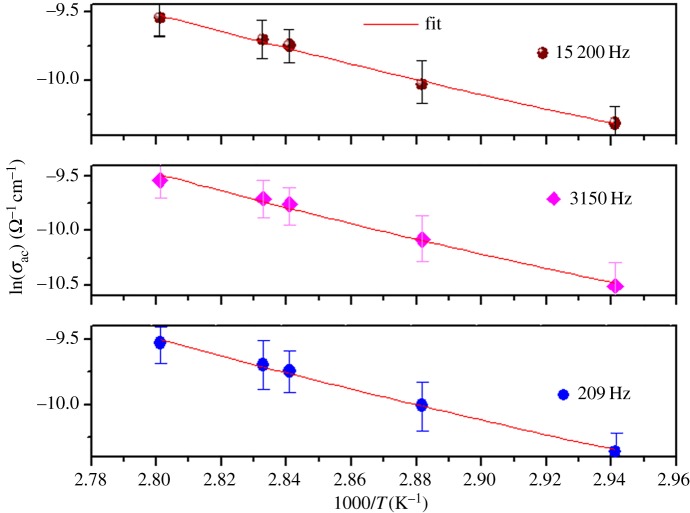
Temperature dependences of *σ*_ac_ at different frequencies of LDSMO.

The ac conductivity of this sample LDSMO can be satisfactorily explained by considering only one conduction mechanism (single polaron). Figure 11 shows the temperature dependence of the ac conductivity in LDSMO. It is clear that the ac conductivity varied exponentially with temperature because the ln(*σ*_ac_) versus 1000/T plots are straight lines. Figure 11 clearly shows that the theoretical calculations fit well with the experimental data. The values of the CBH model parameters have been adjusted to fit the calculated graphs of ln(*σ*_ac_) versus 1000/*T* to the experimental curves. The values of the parameter of the CBH model conduction for LDSMO sample are listed in table 6.

### 3.4. Electric modulus analysis

Formalism of the electric modulus is used to study the electrical relaxation process and is suitable for extracting electrode polarization. The complex electric modulus *M* is defined by the reciprocal of the complex permittivity ε(M=1/ε), which can be described as [48]:

$$M=M^{\prime }+jM^{\prime \prime },$$

where *M^′^* and *M^″^* are the real and imaginary parts of the complex modulus, respectively.

**Figure 12.**
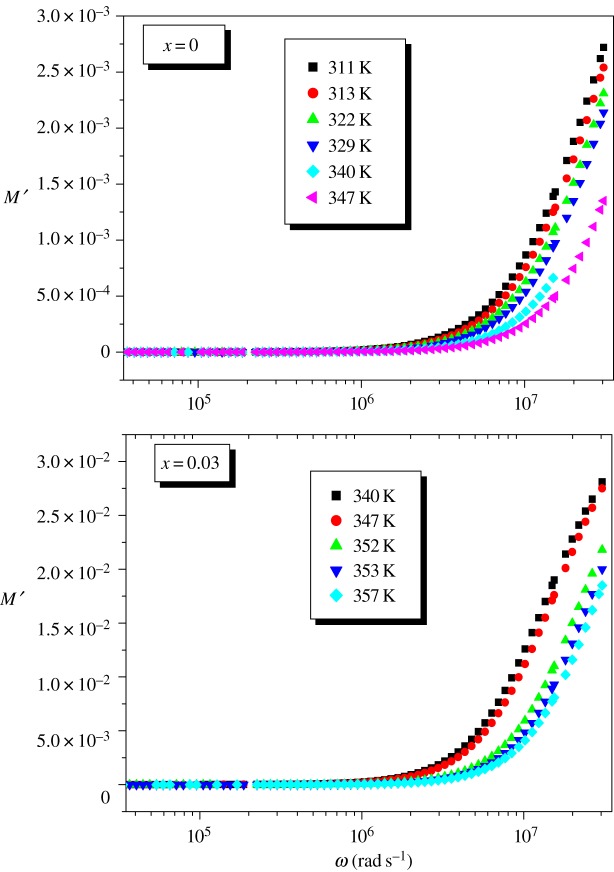
Variation in the real part of the modulus as a function of angular frequency at different temperatures for La_0.7−*x*_Dy*_x_*Sr_0.3_MnO_3_ (*x* = 0.00 and 0.03) compounds.

**Figure 13.**
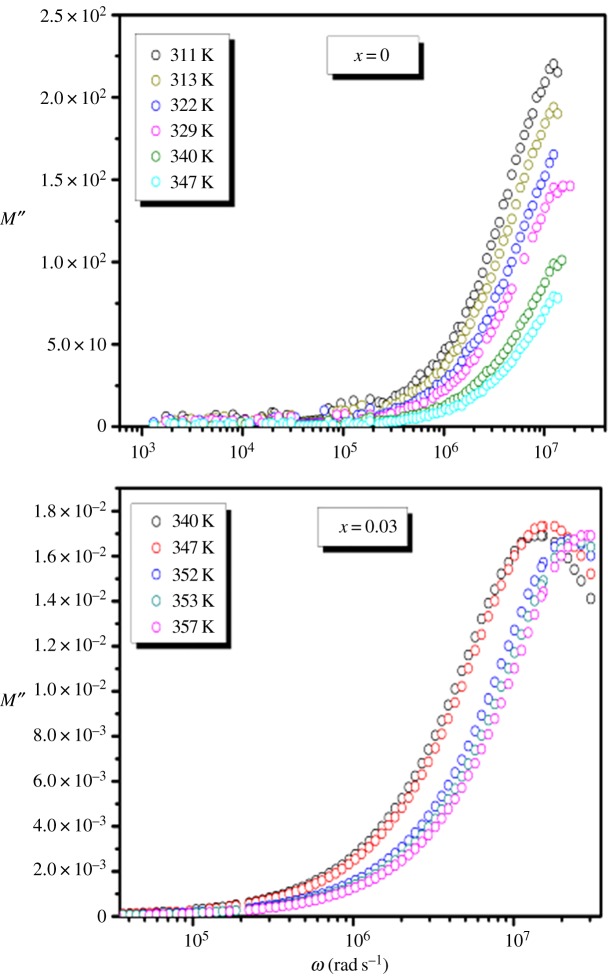
Variation of *M*″ with angular frequency at different temperatures for the La_0.7−*x*_Dy*_x_*Sr_0.3_MnO_3_ (*x* = 0.00 and 0.03) compounds.

Figures 12 and 13 show the real and imaginary parts of the modulus, respectively, at different temperatures for La_0.7−*x*_Dy*_x_*Sr_0.3_MnO_3_ (*x* = 0.00 and 0.03) samples.

For both compounds, figure 12 reveals that the real modulus *M*^′^ is dispersed with frequency. The small value of *M*^′^ in the low frequency region facilitates the migration of ion conduction [49].

Figure 13 shows that *M*^″^ has a single relaxation peak centred at the dispersion region of *M*′ which is well defined for LDSMO oxide and is associated with the grain effect. The left region corresponds to a zone in which the Sr^2+^ ions are mobile over long ranges, whereas, the right one is ascribed to spatially confined ions [50].

The movement of the charge carriers becomes faster as the temperature is increased, leading to a decreased relaxation time and a consequent shift in the *M*″ peak towards higher frequencies. This behaviour suggests that the relaxation is thermally activated and charge carrier hopping is taking place [49].

**Figure 14.**
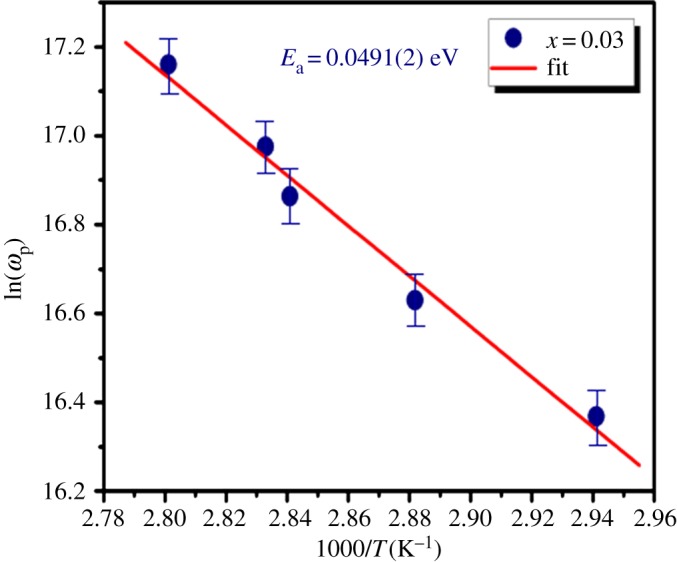
The variation of log(*ω*_max_) as a function of 1000/*T* for LDSMO compound.

The value of the maximum frequency peak *ω*_max_ has been determined in the measured temperature range for the substituted compound LDSMO. The variation of ln (*ω*_max_) as a function of temperature is shown in figure 14. It appears to satisfy the Arrhenius law given by:

3.17 $$\omega_{\max}=\omega_{o}\exp\left(\frac{-E_{a}}{k_{B}T}\right),$$

where *ω*_o_ is the pre-exponential factor and *E*_a_ is the activation energy for the relaxation process.

The activation energy extracted from the slope of the plot is 0.491(2) eV, which is in good agreement with that simulated from the impedance measurement (0.535(7) eV), suggesting that the mobility of the charge carrier is due to a hopping mechanism [51] dominated by the motion of the Sr ions.

## 4. Conclusion

In this work, La_0.7−*x*_Dy*_x_*Sr_0.3_MnO_3_ (*x* = 0.00 and 0.03) compounds were synthesized using the sol–gel method. XRD analysis reveals a structure transition from the rhombohedral system (R-3c) to the orthorhombic one (Pnma) with Dy substitution. One semicircle is observed in the impedance plot indicating the presence of only one relaxation process in these compounds, associated with the bulk effect. The presence of one relaxation peak thermally activated in the modulus spectra confirms the grain contribution to electrical response in these materials. The addition of a small amount (3%) of Dy content in LSMO induces an increase in the activation energy value, deduced from the analysis of the equivalent circuit. We can correlate the activation energy of these materials to the ionic radii of the A site cations in AMnO_3_ perovskite oxide, the mismatch effect *σ*^2^ and the bandwidth *W*. The displacements of the Sr^2+^ ion are probably due to the NSPT mechanism in the tunnel-type cavities along [111] direction in the parent compound La_0.7_Sr_0.3_MnO_3_. However, this model is not applicable in the substituted oxide La_0.67_Dy_0.03_Sr_0.3_MnO_3_. The close value of activation energies obtained from the analyses of M′(0.491(2) eV) and conductivity data (0.535(7) eV) suggest that the mobility of the charge carrier is probably due to a hopping of Sr^2+^ ions in the La_0.67_Dy_0.03_Sr_0.3_MnO_3_ oxide.

In this work, we have deduced that the insertion of a small amount of dysprosium (3%) in the La-site of La_0.7_Sr_0.3_MnO_3_ perovskite oxide can change the mechanism conduction of charge transport.

## Data accessibility

This article does not contain any additional data.
